# Phase I and pharmacokinetic study of the topoisomerase II catalytic inhibitor fostriecin

**DOI:** 10.1038/sj.bjc.6690141

**Published:** 1999-02

**Authors:** R S de Jong, N H Mulder, D R A Uges, D Th Sleijfer, F J P Höppener, H J M Groen, P H B Willemse, W T A van der Graaf, E G E de Vries

**Affiliations:** Department of Medical Oncology, University Hospital Groningen, Groningen, The Netherlands; Department of Pharmacy and Toxicology, University Hospital Groningen, Groningen, The Netherlands; Department of Pulmonary Diseases, University Hospital Groningen, Groningen, The Netherlands; EORTC New Drug Development Office, Amsterdam, The Netherlands

**Keywords:** fostriecin, topoisomerase II, phase I, pharmacokinetics

## Abstract

We conducted a phase I and pharmacokinetic study of the topoisomerase II catalytic inhibitor fostriecin. Fostriecin was administered intravenously over 60 min on days 1–5 at 4-week intervals. Dose was escalated from 2 mg m^−2^day^−1^to 20 mg m^−2^day^−1^in 20 patients. Drug pharmacokinetics was analysed with high performance liquid chromatography with UV-detection. Plasma collected during drug administration was tested in vitro for growth inhibition of a teniposide-resistant small-cell lung cancer (SCLC) cell line. The predominant toxicities were elevated liver transaminases (maximum common toxicity criteria (CTC) grade 4) and serum creatinine (maximum CTC grade 2). These showed only a limited increase with increasing doses, often recovered during drug administration and were fully reversible. Duration of elevated alanine–amino transferase (ALT) was dose-limiting in one patient at 20 mg m^−2^. Other frequent toxicities were grade 1–2 nausea/vomiting, fever and mild fatigue. Mean fostriecin plasma half-life was 0.36 h (initial; 95% CI, 0–0.76 h) and 1.51 h (terminal; 95% CI, 0.41–2.61 h). A metabolite, most probably dephosphorylated fostriecin, was detected in plasma and urine. No tumour responses were observed, but the plasma concentrations reached in the patients were insufficient to induce significant growth inhibition in vitro. The maximum tolerated dose (MTD) has not been reached, because drug supply was stopped at the 20 mg m^−2^dose level. However, further escalation seems possible and is warranted to achieve potentially effective drug levels. Fostriecin has a short plasma half-life and longer duration of infusion should be considered. © 1999 Cancer Research Campaign


*British Journal of Cancer* (1999) **79**(5/6), 882–887
© 1999 Cancer Research Campaign
Article no. bjoc.1998.0141

### Phase I and pharmacokinetic study of the topoisomerase II catalytic inhibitor fostriecin

**RS de Jong[1], NH Mulder[1], DRA Uges[2], DTh Sleijfer[1], FJP Höppener[4], HJM Groen[3], PHB Willemse[1], WTA van der Graaf[1] and EGE de Vries[1]**

Departments of [1]Medical Oncology, [2]Pharmacy and Toxicology, and [3]Pulmonary Diseases, University Hospital Groningen, The Netherlands; [4]EORTC New Drug Development Office, Amsterdam, The Netherlands

**Summary** We conducted a phase I and pharmacokinetic study of the topoisomerase II catalytic inhibitor fostriecin. Fostriecin was administered intravenously over 60 min on days 1–5 at 4-week intervals. Dose was escalated from 2 mg $m^{-2}$ $day^{-1}$ to 20 mg $m^{-2}$ $day^{-1}$ in 20 patients. Drug pharmacokinetics was analysed with high performance liquid chromatography with UV-detection. Plasma collected during drug administration was tested in vitro for growth inhibition of a teniposide-resistant small-cell lung cancer (SCLC) cell line. The predominant toxicities were elevated liver transaminases (maximum common toxicity criteria (CTC) grade 4) and serum creatinine (maximum CTC grade 2). These showed only a limited increase with increasing doses, often recovered during drug administration and were fully reversible. Duration of elevated alanine–amino transferase (ALT) was dose-limiting in one patient at 20 mg $m^{-2}$. Other frequent toxicities were grade 1–2 nausea/vomiting, fever and mild fatigue. Mean fostriecin plasma half-life was 0.36 h (initial; 95% CI, 0–0.76 h) and 1.51 h (terminal; 95% CI, 0.41–2.61 h). A metabolite, most probably dephosphorylated fostriecin, was detected in plasma and urine. No tumour responses were observed, but the plasma concentrations reached in the patients were insufficient to induce significant growth inhibition in vitro. The maximum tolerated dose (MTD) has not been reached, because drug supply was stopped at the 20 mg $m^{-2}$ dose level. However, further escalation seems possible and is warranted to achieve potentially effective drug levels. Fostriecin has a short plasma half-life and longer duration of infusion should be considered.

**Keywords**: fostriecin; topoisomerase II; phase I; pharmacokinetics

Fostriecin (CI-920) is a topoisomerase (topo) II catalytic activity inhibitor (Boritzki et al, 1988) (Figure 1). This novel class of topo II-targeting drugs is considered of potential value for treatment of patients with tumours resistant to classic topo II poisons, including anthracyclines and podophyllotoxins (Cummings and Smyth, 1993). The nuclear enzyme topo II is essential for the regulation of DNA conformation (reviewed in Osheroff et al, 1991). Central in its action is the formation of a complex with DNA. Stabilization of this complex by topo II poisons induces DNA damage and cell death (Froelich-Ammon and Osheroff, 1995). Decreased complex formation due to decreased topo II levels is considered to be an important mechanism of tumour-resistance to topo II poisons (Beck and Danks, 1991). The topo II catalytic inhibitor fostriecin is expected to have increased activity against tumour cells with low topo II levels and this was confirmed in in vitro studies (De Jong et al, 1991). In vitro, fostriecin also inhibited nuclear protein phosphatases involved with cell cycle regulation and histone phosphatases involved with chromosome condensation during mitosis (Roberge et al, 1994; Guo et al, 1995).

In preclinical studies, fostriecin was active against murine leukaemias P388 and L1210 and one of the most active drugs in a human tumour clonogenic assay (Leopold et al, 1984; Scheithauer et al, 1986). Animal experiments showed increased anti-tumour activity with prolonged exposure (Leopold et al, 1984); therefore, a 5-day schedule was chosen for this phase I study. At this schedule the mouse LD10 was 120 mg $m^{-2}$ $day^{-1}$. Because of increased toxicity of fostriecin in dogs compared to mice, a dose below one-third of the one-tenth mouse equivalent LD10, 2 mg $m^{-2}$ $day^{-1}$, was chosen as starting dose in humans (Clinical Brochure Fostriecin, 1991). Histological studies rats showed degenerative changes in renal cortical epithelium and necrosis of lymphoid tissue. In dogs, increased liver transaminases were observed and histologic examinations showed congestion and haemorrhage in several organs, primarily heart and brain, at higher doses.

We also performed an analysis of the human pharmacokinetics of fostriecin and ex vivo experiments to investigate if the plasma drug concentrations achieved were sufficient to inhibit a teniposide-resistant tumour cell line. The maximum tolerated dose (MTD) of fostriecin could not be established because drug supply was stopped prematurely. However, results of this study may provide a basis for continued clinical development of this agent.

*Received 28 October 1997*
*Revised 10 August 1998*
*Accepted 20 October 1998*

*Correspondence to:* EGE de Vries, Department of Internal Medicine, Division of Medical Oncology, University Hospital Groningen, PO Box 30.001, 9700 RB Groningen, The Netherlands

### PATIENTS AND METHODS

### Patient selection, drug administration and evaluation

Patient eligibility criteria were: histologically confirmed diagnosis of a solid tumour, no longer amenable to established forms of treatment; age 18–75 years; Eastern Cooperative Oncology Group (ECOG) performance status ≤ 2; life expectancy ≥ 12 weeks; no

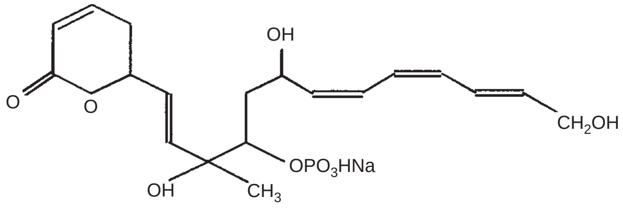

**Figure 1** Chemical structure of fostriecin prior chemo-, immuno- or radiotherapy for at least 4 weeks before study entry; white blood cell (WBC) count ≥ 4000 µl$^{-1}$ and platelet count ≥ 100 000 µl$^{-1}$; bilirubin ≤ 25 µmol l$^{-1}$ and aspartate–amino transferase (AST) and alanine–amino transferase (ALT) within 2.5 times the normal upper limit; normal prothrombin time (PT); creatinine clearance ≥ 60 ml min$^{-1}$. Written informed consent was obtained from all patients. The protocol was approved by the Medical Ethical Committee of the University Hospital Groningen.

Fostriecin was supplied by NCI (Bethesda, MD, USA) as a lyophilized powder and was diluted with 0.9% NaCl to 50 or 100 ml. It was administered as a 60-min intravenous (IV) infusion through an UV-light protected system on days 1–5 at 4-week intervals. The dose was initially escalated according to the Fibonacci scheme, with subsequent dose steps of 2, 4, 6.6, 10 and 12.2 mg m$^{-2}$ day$^{-1}$ and thereafter increased to 20 mg m$^{-2}$ (the protocol was amended during the study to allow more rapid dose escalation). Three patients were entered at each dose level, with a maximum of three additional patients when one out of three experienced dose-limiting toxicity (DLT). When no DLT was observed, or only in one out of six patients, the subsequent group was treated at the next higher dose level. Individual patients were treated until progressive disease for a maximum of 6 courses.

Patients were hospitalized during drug administration. The body temperature and blood pressure were measured four times daily, including a measurement at the start and the end of each infusion. Blood chemistry, including creatine kinase (CK) and urinalysis were performed daily during treatment. During treatment-free intervals patients were evaluated twice-weekly for toxicity. At these evaluations full blood counts, differential, blood chemistry including liver- and renal parameters, CK and urinalysis were performed. In six patients (13 courses) at 10–20 mg m$^{-2}$, PT, fibrinogen, anti-thrombin III and cholinesterase were measured on days 0 and 5 to monitor liver synthesis function.

Toxicities were graded with the NCI common toxicity criteria (CTC). DLT was considered to be grade 4 haematologic or ≥ grade 3 non-haematologic toxicity, or ≥ grade 2 cardiac- or neurotoxicity. After the first two dose levels the protocol was modified to consider ≥ grade 3 renal- and hepatic toxicity as dose-limiting when lasting > 7 days. The MTD was defined as the dose at which not more than one out of three to six patients experienced DLT, with the next higher dose level causing DLT in two or more patients.

### Pharmacokinetics

On day 1 of the first course blood samples (8 ml) were obtained through an IV plastic catheter in the forearm contralateral to the infusion site. The samples were collected in tubes containing heparin sodium (Becton Dickinson Vacutainer Systems Europe, Meylan, France) before infusion, at 30 and 45 min during infusion, at the end of infusion, at 10, 20 and 30 min, and at 1, 1.5, 2, 4, 7 and 17 h after the end of infusion. The samples were immediately centrifuged and the plasma was transferred into polystyrene tubes. Urine was collected immediately before start of infusion, 0–2 h, 2–5 h and 5–18 h after start of infusion. Plasma and urine samples were stored until analysis at –80°C.

Fostriecin plasma concentrations were determined with a high performance liquid chromatography (HPLC) and UV-spectrophotometric detection according to Pillon et al (1994), with modifications to decrease the lower limit of quantification (LLQ). Sulfaquinoxaline (Sigma, Zwijndrecht, The Netherlands) was used as internal standard. Plasma samples were thawed, 0.5 ml placed in a brown glass tube, and 100 µl 0.067 M phosphate buffer (pH 6.9) and 100 µl sulfaquinoxaline (5 mg l$^{-1}$ in ultra pure water) were added. Then 1.5 ml acetonitrile (HPLC grade, Rathburn, Walkerburn, UK) was added and the mixture vortexed 30 s and centrifuged 5 min at 1500 ***g***. The supernatant was transferred to a second glass tube, 4 ml dichloromethane (Merck, Darmstadt, Germany) was added and the tube vortexed and centrifuged as above. Then 300 µl of the aqueous upper layer was transferred to a third tube and any traces of dichloromethane were evaporated in 30 min at 40°C under vacuum. Of the residual solution, 50 µl was injected onto a Nucleosil 100-3C18 HPLC column, 100 × 4.6 mm I.D. (Bouma and Uges, 1980). The mobile phase was an acetonitrile plus phosphate buffer (7.5 + 92.5 v/v) solution (final pH 7.1), at a flow rate of 1.8 ml min$^{-1}$. An UV–HPLC-detector (Spectroflow 757; ABI Analytical Kratos Division, Ramsey, NJ, USA) at 268 nm was used for detection. Concentrations were calculated on a calibration curve using spiked pool human plasma which had been handled the same way at the same time. Extraction of fostriecin was 53.7 ± 9.1% (at 287 µg l$^{-1}$, $n$ = 6). The calibration curves were linear over the range 0.05–2 mg l$^{-1}$ with a correlation coefficient ($r$) ≥ 0.999. The LLQ was 50 µg l$^{-1}$; within-run precision at 390 µg l$^{-1}$ fostriecin was 4.5% ($n$ = 6) with an accuracy of 99%.

For analysis of fostriecin in urine, 100 µl urine was mixed with 50 µl sulfaquinoxaline (5 mg l$^{-1}$ in water) and 100 µl ultra pure water. Then 750 µl phosphate buffer (0.067 M, pH 6.9) was added and after vortexing again, 50 µl was injected onto the HPLC column. The calibration curves were linear from 0.5 to 5 mg l$^{-1}$ with $r$ ≥ 0.999. The LLQ in urine was 0.514 mg l$^{-1}$; within-run precision at 2.57 mg l$^{-1}$ fostriecin was 2.2% ($n$ = 6) with 97.3% accuracy.

Pharmacokinetic parameters were calculated using the MW/PHARM computer package (MediWare, Groningen, The Netherlands) (Proost and Meijer, 1992).

### In vitro analysis for anti-tumour activity of plasma specimens

Plasma samples were assayed for in vitro cytotoxicity against the small-cell lung cancer (SCLC) cell line GLC4/VM$_{20x}$. This teniposide (VM26)-resistant cell line had acquired 20-fold resistance compared to the parental cell line GLC4, with a 50% decrease of topo IIα, but no decrease of topo IIβ, compared to GLC4. Both cell lines had no P-glycoprotein overexpression. GLC4/VM$_{20x}$ was 1.7 times more sensitive to fostriecin than GLC4 (Withoff et al, 1996). The cell lines were cultured in Roswell Park Memorial Institute (RPMI)-1640 medium (Gibco, Paisley, UK) and 10% fetal calf serum (Sanbio, Uden, The Netherlands), without teniposide for at least 1 month. With a microtiterwell tetrazolium assay (Timmer-Bosscha et al, 1989), the growth-inhibitory activity of plasma samples collected at the time of maximal fostriecin plasma concentration ($T_{max}$) was determined. Cells (7.5 × 10$^4$ ml$^{-1}$) were incubated

© Cancer Research Campaign 1999

4 days in Ham F12 medium plus Dulbecco's modified Eagle medium (1:1) (Life Technologies, Breda, The Netherlands) and 40% patient plasma (higher plasma concentrations caused an unacceptable background signal). Growth of GLC4/$VM_{20x}$ exposed to plasma collected at $T_{max}$ was compared to growth in medium containing 40% plasma from the same patient collected before treatment and expressed as percentage. Plasma samples from two patients at 6.6 mg $m^{-2}$ ($n$ = 2), two at 12.2 mg $m^{-2}$ ($n$ = 2) and two at 20 mg $m^{-2}$ ($n$ = 4) were tested. Positive controls were included for all patient samples by adding fostriecin to drug-free patient plasma. A reference growth curve of GLC4/$VM_{20x}$ exposed to 0.5, 1, 2, 3, 4, 5, 7.5, 15 and 30 μM fostriecin and cultured in medium with 40% pooled human plasma derived from 20 healthy volunteers, was included with each separate assay. Experiments with patient samples were performed once in quadruplicate and results expressed as mean values ± SD. A mean reference curve was based on results of five separate experiments, each in quadruplicate.

### RESULTS

Patient characteristics are listed in Table 1. Baseline liver transaminases were slightly elevated in 10 of 12 patients with liver metastases (AST 42–52 U $l^{-1}$; ALT 34–69 U $l^{-1}$).

The predominant laboratory abnormalities observed during fostriecin administration (considered at least possibly drug-related) are listed in Table 2. Elevated liver transaminases (ALT more than AST) were observed already at the first dose level and were the only toxicities that reached grade 3 or 4. No concurrent elevations of CK were observed, which excluded muscle toxicity. There was no relationship between presence of liver metastases and grade 3–4 liver toxicity (Fisher's exact test: $P$ = 0.65). ALT elevations were highly variable and did not clearly increase with repeated administration. ALT levels in patients with elevations ≥ grade 3 during courses 1 or 2 are depicted in Figure 2. ALT peaked between days 2 and 12 per course (median: day 2.5) and median duration of ≥ grade 3 ALT was 3.5 days (range: 1–9 days). Grade 3 ALT persisted > 7 days, and was therefore considered dose-limiting, in one patient at 20 mg $m^{-2}$. However, no MTD could be defined because drug supply was stopped at this dose level. In all patients ALT and AST elevations resolved within 3 weeks after drug administration. Reversible increases of bilirubin were observed in four patients (highest value 43 μmol $l^{-1}$, conjugated bilirubin 18 μmol $l^{-1}$). PT, fibrinogen, anti-thrombin III and cholinesterase levels were not affected.

Elevated serum creatinine was the second most common toxicity. Serum creatinine peaked between days 2 and 6 (median: day 3). Transient proteinuria was observed during drug administration at doses ≥ 10 mg $m^{-2}$ (grade 1 in seven patients and grade 2 in

**Table 1** Patient characteristics ($n$ = 20)

| | |
|---|---|
| Median age in years (range) | 50 (23–71) |
| | Number |
| Male/female | 13/7 |
| Primary site | |
| Colorectal cancer | 10 |
| Non-small cell lung cancer | 4 |
| Other[a] | 6 |
| ECOG performance score | |
| 0 | 13 |
| 1 | 5 |
| 2 | 2 |
| Prior therapy | |
| None | 5 |
| Chemotherapy alone | 7 |
| Chemotherapy and radiotherapy | 8 |
| Number of patients with metastatic disease[b] | |
| At one site | 4 |
| At multiple sites | 9 |

[a]Breast cancer, melanoma, sarcoma, medullary thyroid carcinoma, bile duct carcinoma and adenocarcinoma of unknown primary. [b]Including liver metastases in 12 patients.

**Table 2** Predominant laboratory abnormalities during fostriecin administration (first course/all courses)

| Dose level (mg $m^{-2}$) | Number of patients/courses (full dose) | CTC-grade AST | | | | CTC-grade ALT | | | | CTC-grade GGT[b] | | | |
|---|---|---|---|---|---|---|---|---|---|---|---|---|---|
| | | 1 | 2 | 3 | 4 | 1 | 2 | 3 | 4 | 1 | 2 | 3 | 4 |
| 2 | 4/8 | 2/2[a] | – | – | – | 2/6 | 1/1 | – | – | 0/2 | 1/2 | – | – |
| 4 | 5/11 | 3/7 | 1/1 | 0/1 | – | 4/8 | 0/1 | 1/2 | – | 1/1 | 0/0 | 1/4 | – |
| 6.6 | 3/4 | 0/0 | 1/1 | 1/2 | – | 2/2 | 1/1 | 0/1 | – | 0/0 | 0/0 | 1/1 | – |
| 10 | 3/7 | 0/3 | 1/1 | – | – | 0/0 | 0/3 | 2/2 | – | 2/6 | 0/0 | 1/1 | – |
| 12.2 | 3/8 | 0/0 | 0/3 | 3/4 | – | 0/0 | 0/1 | 2/6 | 1/1 | 1/2 | 1/2 | 1/2 | – |
| 20 | 2/4 | 0/0 | 0/1 | 1/2 | – | 0/0 | 1/2 | 1/2 | – | 1/2 | 0/0 | 1/2 | – |

| Dose level (mg $m^{-2}$) | Number of patients/courses (full dose) | CTC-grade ALP[c] | | | | CTC-grade bilirubin[d] | | | CTC-grade creatinine | | | |
|---|---|---|---|---|---|---|---|---|---|---|---|---|
| | | 1 | 2 | 3 | 4 | 2 | 3 | 4 | 1 | 2 | 3 | 4 |
| 2 | 4/8 | 1/2 | – | – | – | – | – | – | 2/4 | – | – | – |
| 4 | 5/11 | 3/3 | 0/1 | – | – | – | – | – | 3/7 | – | – | – |
| 6.6 | 3/4 | 0/3 | 0/1 | – | – | 1/1 | – | – | 1/1 | – | – | – |
| 10 | 3/7 | 1/1 | – | – | – | – | – | – | 2/3 | 1/4 | – | – |
| 12.2 | 3/8 | 1/3 | 0/1 | – | – | 2/6 | 0/1 | – | 1/5 | 2/3 | – | – |
| 20 | 2/4 | 2/2 | – | – | – | – | – | – | 1/1 | 1/2 | – | – |

[a]Number of patients developing toxicity in the first course/number of courses causing toxicity for all courses given at full dose. [b]Gamma-glutamyl transpeptidase. [c]Alkaline phosphatase. [d]The CTC classification starts at grade 2 for abnormal bilirubin.

 *© Cancer Research Campaign 1999*

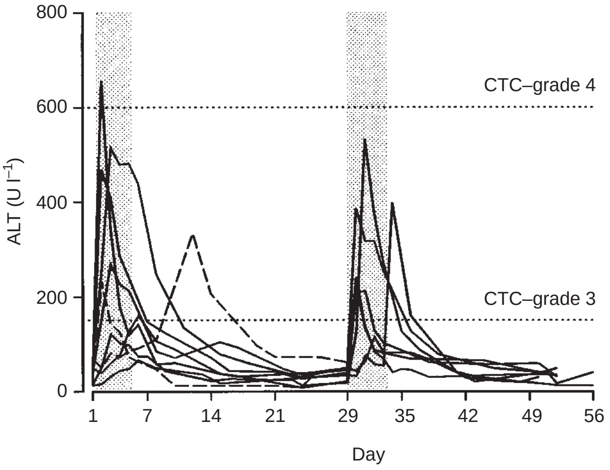

**Figure 2** ALT serum levels during courses 1 and 2 in patients with ≥ grade 3 ALT elevation during at least one course ($n$ = 9). Two patients received only 1 course (dashed lines). Shaded areas represent days of fostriecin administration one). Microscopic haematuria (= grade 1) was recorded in seven patients at doses ≥ 6.6 mg m$^{-2}$. All renal toxicities resolved within 1–2 weeks after the last drug dose of each course.

No leuco- or neutropenia was observed. Reversible thrombocytopenia (grade 2) developed in one patient at 20 mg m$^{-2}$. Fourteen patients developed grade 1–2 anaemia.

The main symptomatic toxicities were grade 1–2 nausea/vomiting and transient increases in body temperature. These were observed from the first dose level and there were no obvious dose–response relationships. Nausea/vomiting were observed in 11 patients during course 1 and during 33 of the 42 courses at full dose. The patients responded well to metoclopramide, although this was not given prophylactically. In 17 patients body temperature increased occasionally (in 14 during course 1), in particular immediately or a few hours after drug administration on day 1 and/or day 2. Fever > 38°C (≥ grade 2) was recorded during 11 courses in six patients and > 39°C in one. Blood cultures performed in the latter patient were negative. No antipyretics were given. Eight patients reported mild fatigue (grade 1–2) during drug administration and the week thereafter.

In seven patients hyperglycaemia grade 1–2 and/or hypoglycaemia grade 1 was observed. One patient was known with diabetes and insulin dose was adjusted; in the others blood glucose normalized spontaneously. The other grade 2 toxicities included LDH increases in three patients, and hyponatraemia and oedema each in one patient (see below).

All toxicities, except anaemia, resolved within the 3-week treatment-free interval and all subsequent courses could be administered as scheduled. The fostriecin dose was reduced 50% in two patients: in one patient at 4 mg m$^{-2}$ after course 1 because of grade 3 liver toxicity (during the 3 subsequent courses this did not exceed grade 2) and in one patient at 20 mg m$^{-2}$ after course 2 because of grade 2 oedema with hypoalbuminaemia (28 g l$^{-1}$) and transient proteinuria (maximum: 3.3 g 24 h$^{-1}$). During the subsequent 2 courses serum albumin was never below 31 g l$^{-1}$ and only slight peripheral oedema remained.

No tumour responses were observed in any patient (10 had measurable disease).

### Pharmacokinetics

Plasma pharmacokinetics (see Table 3) could best be described by a two-compartment model. A close linear association of drug dose with $C_{max}$ (Pearson's $r$ = 0.95, $P$ < 0.001) and with AUC ($r$ = 0.89, $P$ < 0.001) indicated linear pharmacokinetics within the investigated dose range. Plasma levels decreased rapidly (Figure 3) and in only one patient was fostriecin detectable for more then 7 h after infusion. Pharmacokinetic parameters were calculated for the two-compartment model: mean plasma half-life 0.36 h ($t_{1/2\alpha}$; 95% CI, 0–0.76 h) and 1.51 h ($t_{1/2\beta}$; 95% CI, 0.41–2.61 h); mean Cl (apparent total body clearance) 2.90 l h$^{-1}$ m$^{-2}$; (= 48.3 ml min$^{-1}$ m$^{-2}$; 95% CI, 2.24–3.57 l h$^{-1}$ m$^{-2}$); mean residence time (MRT) 1.19 h (95% CI, 0.41–1.97 h); mean volume of distribution ($V_d$)

**Table 3** Fostriecin plasma pharmacokinetic parameters after a 60 min IV administration of fostriecin on day 1 of the first course

| Patient no. | Dose (mg m$^{-2}$) | $C_{max}$ (mg l$^{-1}$) | AUC (mg l$^{-1}$ h$^{-1}$) | $t_{1/2\alpha}$ (h) | $t_{1/2\beta}$ (h) | Cl (l h$^{-1}$ m$^{-2}$) | MRT (h) | $V_d$ (l m$^{-2}$) |
|---|---|---|---|---|---|---|---|---|
| 1[a] | 2 | 0.495 | 0.739 | 0.60 | – | 5.14 | 0.86 | 4.44 |
| 3[a] | 2 | 0.597 | 0.810 | 0.54 | – | 2.40 | 0.77 | 1.86 |
| 4[a] | 2 | 0.371 | 0.468 | 0.35 | – | 4.11 | 0.51 | 2.10 |
| 5[a] | 4 | 1.163 | 1.360 | 0.50 | – | 2.81 | 0.72 | 2.03 |
| 6[a] | 4 | 0.956 | 1.167 | 0.46 | – | 3.05 | 0.67 | 2.03 |
| 7 | 4 | 0.940 | 1.486 | 0.26 | 2.03 | 2.31 | 1.93 | 6.76 |
| 11 | 6.6 | 1.403 | 1.647 | 0.09 | 0.47 | 4.06 | 0.44 | 2.75 |
| 12 | 6.6 | 1.861 | 2.450 | 0.25 | 1.22 | 2.63 | 0.63 | 4.64 |
| 13 | 10 | 2.242 | 3.350 | 1.67 | 0.93 | 2.97 | 0.92 | 3.98 |
| 15 | 10 | 2.446 | 3.768 | 0.30 | 1.39 | 2.66 | 1.01 | 5.34 |
| 16 | 12.2 | 3.201 | 4.230 | 0.22 | 1.17 | 2.84 | 0.94 | 4.80 |
| 18 | 12.2 | 2.787 | 5.324 | 0.46 | 5.10 | 2.32 | 3.61 | 17.09 |
| 19[b] | 20 | 3.847 | 4.446 | 0.03 | 0.54 | 4.52 | 0.38 | 3.39 |
| 20 | 20 | 6.354 | 9.965 | 0.01 | 0.76 | 1.83 | 0.83 | 2.01 |

$C_{max}$ = maximum plasma concentration, observed values are given; Cl = apparent total body clearance; MRT = mean residence time; $V_d$ = volume of distribution. [a]One-compartment pharmacokinetic model because of low plasma-concentrations. In patient nos 7–20 a two-compartment model was used ($r^2$ ≥ 0.977). [b]Sampling performed on day 1 of course 2.

© Cancer Research Campaign 1999

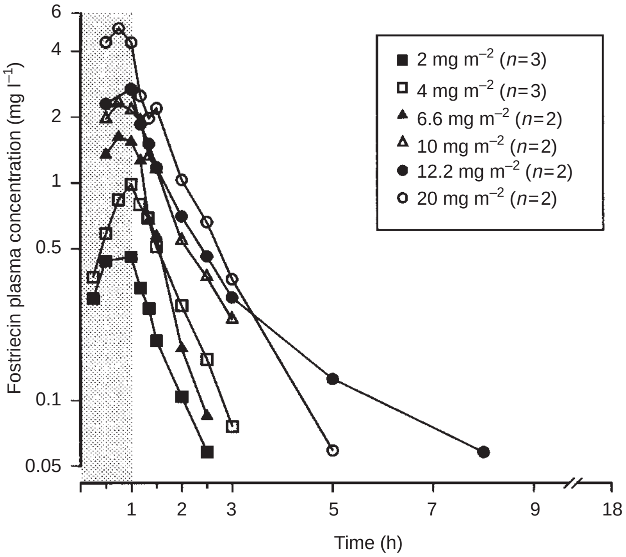

**Figure 3** Mean fostriecin plasma-concentrations during (shaded area) and after drug infusion at the various dose levels (semilogarithmic scale)

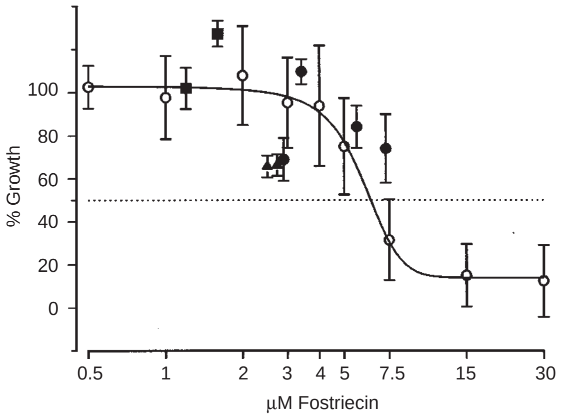

**Figure 4** Growth inhibition of GLC4/$VM_{20x}$ exposed to serial dilutions of fostriecin in culture medium containing pooled human plasma (○: mean of 5 experiments; error bars, SD) and growth inhibition of GLC4/$VM_{20x}$ cultured in medium containing plasma from patients collected at $T_{max}$ following fostriecin administration (fostriecin doses: ■ = 6.6 mg m$^{-2}$, ▲ = 12.2 mg m$^{-2}$, ● = 20 mg m$^{-2}$; error bars, SD)

5.64 l m$^{-2}$ (95% CI, 2.16–9.11 l m$^{-2}$). In one patient (no. 18) an exceptionally large $V_d$ was observed. Fostriecin was detectable in urine of patient nos 12–20. Renal excretion accounted for 14.6% (mean; 95% CI, 10–17.6%) of drug elimination in these patients.

A second compound, with a retention time approximately 25 min after fostriecin, was detected in plasma and urine of patients who received ≥ 12.2 mg m$^{-2}$ fostriecin. A similar chromatographic peak was observed after incubation of fostriecin in water with alkaline phosphatase (Sigma, Zwijndrecht, The Netherlands). This indicated that the second compound was similar to dephosphorylated fostriecin.

### In vitro antitumour activity of plasma samples

The mean $IC_{50}$ for fostriecin of GLC4/$VM_{20x}$ cultured in medium with 40% human plasma was 5.9 μM (95% CI, 5.3–6.5 μM). When exposed to patient samples obtained at $T_{max}$, at most 34% growth-inhibition of GLC4/$VM_{20x}$ was observed (Figure 4). Results obtained with patient plasma incubated with fostriecin ex vivo were compatible with the reference curves of GLC4/$VM_{20x}$. This excluded that fostriecin was inactivated by other plasma components.

### DISCUSSION

This phase I study showed that repeated daily IV fostriecin administration is possible in doses up to at least 20 mg m$^{-2}$ day$^{-1}$ for 5 days. At 20 mg m$^{-2}$ dose-limiting liver toxicity was observed in one patient. Drug supply was thereafter stopped by NCI, because 8% related (organic) impurities were detected in the fostriecin batches with a new detection method. This precluded definition of the MTD. The impurities were detected with HPLC at ± 1 mg ml$^{-1}$ fostriecin. However, the clinical samples analysed in our study contained a 150-fold lower drug concentration and no impurities were detected in plasma and urine of our patients.

The observed toxicity pattern was quite different from that of most conventional anti-tumour agents. Liver and renal toxicities prevailed and there was almost no haematologic toxicity. In contrast, in preclinical studies of fostriecin in rodents, haematologic toxicity was common, alongside hepatic and renal disturbances (Susick et al, 1990). Similar increases of transaminases were observed after fostriecin administration in dogs and rabbits (Clinical Brochure Fostriecin, 1991; Pillon et al, 1994). We did not find signs of compromised liver synthesis function. Because the liver toxicities were asymptomatic and most often declined despite continued administration, criteria for dose-limiting toxicity were modified after the second dose level to allow grade 3–4 liver toxicities lasting ≤ 7 days. We further analysed renal toxicity with isotope studies to measure the glomerular filtration rate and renal plasma flow in a subset of eight patients. These data, which confirmed the reversibility of the renal toxicity, were published separately (De Jong et al, 1998).

There was only a limited increase of toxicity with increasing doses. Strikingly, elevated transaminases and serum creatinine most often peaked before or on day 3. This implies that drug administration for another 2–3 days did not result in a further increase of toxicity. Because the reduced folate carrier is responsible for cellular fostriecin uptake, depletion of this carrier might explain this observation (Fry et al, 1984).

Pharmacokinetic analysis showed a rapid decrease of plasma levels after infusion. A similar short half-life had been observed in rabbits (Pillon et al, 1994). Only 15% of the drug was excreted with urine. Therefore, the major part of the hydrophillic fostriecin is expected to be metabolized, or excreted with faeces. We detected a metabolite in plasma and urine at higher dose levels, which was most probably dephosphorylated fostriecin. Dephosphorylation of fostriecin is an important factor to consider, because the intact phosphate ester bond has been shown necessary for cellular uptake and anti-tumour activity (Fry et al, 1984; Leopold et al, 1984). Other investigators reported a metabolite that was not dephosphorylated fostriecin and was also not recovered from urine (Schilsky et al, 1994).

We investigated if plasma levels obtained in vivo could be related to in vitro effects of fostriecin on the growth of a human SCLC cell line, GLC4/$VM_{20x}$. This cell line was selected because of its resistance to the topo II poison teniposide due to low topo II protein levels. Fostriecin is, in particular, expected to be of potential value in treatment of cancers with this type of drug-resistance

 *© Cancer Research Campaign 1999*

against topo II poisons. Our results showed that to reach a significant in vitro effect, i.e. > 50% growth-inhibition, higher concentrations than those obtained in vivo in this study are required. These data should be interpreted with caution because of differences in drug behaviour in vitro and in vivo. Extrapolation to the in vivo situation is also complicated by the fact that plasma had to be diluted 2.5-fold for cell culture experiments. However, a comparison of results obtained in vivo with the plasma samples to the reference curve of GLC4/$VM_{20x}$ indicates that significant in vitro growth-inhibition can be expected when dose is further escalated by one or two steps of 50%.

In conclusion, further dose escalation will be necessary to define the MTD of fostriecin. Based on in vitro investigations with a fostriecin-sensitive SCLC model, clinical activity might be expected at doses of 30–40 mg $m^{-2}$. The toxicities encountered over the present investigated dose range, and in particular the limited progression with increasing doses, suggest that dose escalation to this level would be feasible. For evaluation of efficacy of fostriecin at these doses, patients with potentially sensitive tumours should be selected. The pharmacokinetic data from the present study indicate that the dose schedule should be reconsidered, with particular attention to the feasibility of prolonged infusion because of fostriecin's very short half-life. Continued research on the novel class of topo II catalytic inhibitors, of which fostriecin is a representative, is warranted because of the limited therapeutic options in patients with drug-resistant tumours.

### ACKNOWLEDGEMENTS

We are indebted to P Bouma and EAM Slijfer, of the Department of Pharmacy and Toxicology at the University Hospital Groningen, for their expert technical assistance with the pharmacological analysis.

### REFERENCES

Beck WT and Danks MK (1991) Mechanisms of resistance to drugs that inhibit DNA topoisomerases. *Sem Cancer Biol* **2**: 235–244

Boritzki TJ, Wolfard TS, Besserer JA, Jackson RC and Fry DW (1988) Inhibition of type II topoisomerase by fostriecin. *Biochem Pharmacol* **37**: 4063–4068

Bouma P and Uges DRA (1980) Preparation of highly efficient columns for high-performance liquid chromatography. In: *The Serum Concentration of Drugs, International Congress Series 501*, Merkus FWHM (ed), pp 278–280. Excerpta Medica: Amsterdam

Clinical Brochure Fostriecin (1991) Bethesda, MD, USA, National Cancer Institute

Cummings J and Smyth JF (1993) DNA topoisomerase I and II as targets for rational design of new anticancer drugs. *Ann Oncol* **4**: 533–543

De Jong S, Zijlstra JG, Mulder NH and De Vries EGE (1991) Lack of cross-resistance to fostriecin in a human small-cell lung carcinoma cell line showing topoisomerase II-related drug resistance. *Cancer Chemother Pharmacol* **28**: 461–464

De Jong RS, De Vries EGE, Meijer S, De Jong PE and Mulder NH (1998) Renal toxicity of the anticancer drug fostriecin. *Cancer Chemother Pharmacol* **42**: 160–164

Froehlich-Ammon SJ and Osheroff N (1995) Topoisomerase poisons: harnessing the dark side of enzyme mechanism. *J Biol Chem* **270**: 21429–21432

Fry DW, Besserer JA and Boritzki TJ (1984) Transport of the anti-tumour antibiotic CI-920 into L1210 leukemia cells by the reduced folate carrier system. *Cancer Res* **44**: 3366–3370

Guo XW, Thng JPH, Swank RA, Anderson HJ, Tudan C, Bradbury EM and Roberge M (1995) Chromosome condensation induced by fostriecin does not require p34(cdc2) kinase activity and histone H1 hyperphosphorylation, but is associated with enhanced histone H2A and H3 phosphorylation. *EMBO J* **14**: 976–985

Leopold WR, Shillis JL, Mertus AE, Nelson JM, Roberts BJ and Jackson RC (1984) Anticancer activity of the structurally novel antibiotic CI-920 and its analogues. *Cancer Res* **44**: 1928–1932

Osheroff N, Zechiedrich EL and Gale KC (1991) Catalytic function of DNA topoisomerase II. *BioEssays* **13**: 269–275

Pillon L, Moore MJ and Thiessen JJ (1994) Determination of fostriecin pharmacokinetics in plasma using high-pressure liquid chromatography assay. *Ther Drug Monit* **16**: 186–190

Proost JH and Meijer DKF (1992) MW/PHARM, an integrated software package for drug dosage regimen calculation and therapeutic drug monitoring. *Comput Biol Med* **22**: 155–160

Roberge M, Tudan C, Hung SM, Harder KW, Jirik FR and Anderson H (1994) Antitumour drug fostriecin inhibits the mitotic entry checkpoint and protein phosphatases 1 and 2A. *Cancer Res* **54**: 6115–6121

Scheithauer W, Von Hoff DD, Clark GM, Shillis JL and Elslager EF (1986) In vitro activity of the novel antitumour antibiotic fostriecin (CI-920) in a human tumour cloning assay. *Eur J Cancer Clin Oncol* **22**: 921–926

Schilsky RL, Ramirez J, Wilson K, Vokes E, Kobayashi K, Berezin F, Wulff W and Ratain MJ (1994) Phase I clinical and pharmacological study of fostriecin in patients with advanced cancer. *Proc Am Soc Clin Oncol* **13**: 321

Susick RL, Hawkins KL and Pegg DG (1990) Preclinical toxicological evaluation of fostriecin, a novel anticancer antibiotic, in rats. *Fundam Appl Toxicol* **15**: 258–269

Timmer-Bosscha H, Hospers GAP, Meijer C, Mulder NH, Muskiet FAJ, Martini IA, Uges DRA and De Vries EGE (1989) Influence of docosahexaenoic acid on cisplatin resistance in a human small cell lung carcinoma cell line. *J Natl Cancer Inst* **81**: 1069–1075

Withoff S, De Vries EGE, Keith WN, Nienhuis EF, Van der Graaf WTA, Uges DRA and Mulder NH (1996) Differential expression of DNA Topoisomerase IIα and β in P-gp and MRP negative VM26, m-AMSA and mitoxantrone resistant sublines of the human SCLC cell line $GLC_4$. *Br J Cancer* **74**: 1869–1876

© Cancer Research Campaign 1999

## References

[PDF_00553] Beck W. T., Danks M. K. (1991). Mechanisms of resistance to drugs that inhibit DNA topoisomerases.. Semin Cancer Biol.

[PDF_00555] Boritzki T. J., Wolfard T. S., Besserer J. A., Jackson R. C., Fry D. W. (1988). Inhibition of type II topoisomerase by fostriecin.. Biochem Pharmacol.

[PDF_00562] Cummings J., Smyth J. F. (1993). DNA topoisomerase I and II as targets for rational design of new anticancer drugs.. Ann Oncol.

[PDF_00571] Froelich-Ammon S. J., Osheroff N. (1995). Topoisomerase poisons: harnessing the dark side of enzyme mechanism.. J Biol Chem.

[PDF_00573] Fry D. W., Besserer J. A., Boritzki T. J. (1984). Transport of the antitumor antibiotic Cl-920 into L1210 leukemia cells by the reduced folate carrier system.. Cancer Res.

[PDF_00576] Guo X. W., Th'ng J. P., Swank R. A., Anderson H. J., Tudan C., Bradbury E. M., Roberge M. (1995). Chromosome condensation induced by fostriecin does not require p34cdc2 kinase activity and histone H1 hyperphosphorylation, but is associated with enhanced histone H2A and H3 phosphorylation.. EMBO J.

[PDF_00581] Leopold W. R., Shillis J. L., Mertus A. E., Nelson J. M., Roberts B. J., Jackson R. C. (1984). Anticancer activity of the structurally novel antibiotic Cl-920 and its analogues.. Cancer Res.

[PDF_00584] Osheroff N., Zechiedrich E. L., Gale K. C. (1991). Catalytic function of DNA topoisomerase II.. Bioessays.

[PDF_00586] Pillon L., Moore M. J., Thiessen J. J. (1994). Determination of fostriecin pharmacokinetics in plasma using high-pressure liquid chromatography assay.. Ther Drug Monit.

[PDF_00589] Proost J. H., Meijer D. K. (1992). MW/Pharm, an integrated software package for drug dosage regimen calculation and therapeutic drug monitoring.. Comput Biol Med.

[PDF_00592] Roberge M., Tudan C., Hung S. M., Harder K. W., Jirik F. R., Anderson H. (1994). Antitumor drug fostriecin inhibits the mitotic entry checkpoint and protein phosphatases 1 and 2A.. Cancer Res.

[PDF_00595] Scheithauer W., Von Hoff D. D., Clark G. M., Shillis J. L., Elslager E. F. (1986). In vitro activity of the novel antitumor antibiotic fostriecin (CI-920) in a human tumor cloning assay.. Eur J Cancer Clin Oncol.

[PDF_00601] Susick R. L., Hawkins K. L., Pegg D. G. (1990). Preclinical toxicological evaluation of fostriecin, a novel anticancer antibiotic, in rats.. Fundam Appl Toxicol.

[PDF_00604] Timmer-Bosscha H., Hospers G. A., Meijer C., Mulder N. H., Muskiet F. A., Martini I. A., Uges D. R., de Vries E. G. (1989). Influence of docosahexaenoic acid on cisplatin resistance in a human small cell lung carcinoma cell line.. J Natl Cancer Inst.

[PDF_00608] Withoff S., de Vries E. G., Keith W. N., Nienhuis E. F., van der Graaf W. T., Uges D. R., Mulder N. H. (1996). Differential expression of DNA topoisomerase II alpha and -beta in P-gp and MRP-negative VM26, mAMSA and mitoxantrone-resistant sublines of the human SCLC cell line GLC4.. Br J Cancer.

[PDF_00568] de Jong R. S., de Vries E. G., Meijer S., de Jong P. E., Mulder N. H. (1998). Renal toxicity of the anticancer drug fostriecin.. Cancer Chemother Pharmacol.

[PDF_00564] de Jong S., Zijlstra J. G., Mulder N. H., de Vries E. G. (1991). Lack of cross-resistance to fostriecin in a human small-cell lung carcinoma cell line showing topoisomerase II-related drug resistance.. Cancer Chemother Pharmacol.

